# Exosomal circRELL1 serves as a miR-637 sponge to modulate gastric cancer progression via regulating autophagy activation

**DOI:** 10.1038/s41419-021-04364-6

**Published:** 2022-01-13

**Authors:** Huaiming Sang, Weifeng Zhang, Lei Peng, Shuchun Wei, Xudong Zhu, Keting Huang, Jiajia Yang, Meihong Chen, Yini Dang, Guoxin Zhang

**Affiliations:** 1 Department of Gastroenterology, The First Affiliated Hospital of Nanjing Medical University, Nanjing, 210029 China; 2 Department of Gastroenterology, Renmin Hospital of Wuhan University, Wuhan, Hubei Province China; 3 Key Laboratory of Hubei Province for Digestive System Disease, Wuhan, Hubei Province, 430060 China

**Keywords:** Gastric cancer, Gastric cancer

## Abstract

Circular RNAs (circRNAs) play a vital role in the occurrence and development of tumors, including gastric cancer (GC). However, there are still many circRNAs related to GC whose functions and molecular mechanisms remain undetermined. Herein, we discover circRNA RELL1, which has not been investigated in GC, and it is markedly downregulated in GC tissues, which is related with poor prognosis, more pronounced lymph node metastasis and poor TNM stage. After confirming the circular structure of circRELL1, we found that circRELL1 could block cell proliferation, invasion, migration, and anti-apoptosis in patients with GC by a series of in vivo and in vitro function-related studies. Further mechanism investigation demonstrated that circRELL1 could sponge miR-637 and indirectly unregulated the expression of EPHB3 via modulating autophagy activation in GC. Additionally, circRELL1 can be transmitted by exosomal communication, and exosomal circRELL1 suppressed the malignant behavior of GC in vivo and in vitro. Taken together, this study elucidates the suppressive roles of circRELL1/miR-637/EPHB3 axis through autophagy activation in GC progression, inspiring for further understanding of the underlying molecular mechanisms of GC and providing a promising novel diagnostic circulating biomarker and therapeutic target in GC.

## Introduction

Gastric cancer (GC) incidence in the world is the fifth, ranking third in mortality associated with cancer, the second most common malignant tumors in China [1, 2]. Despite improved surgical approaches and molecular targeting therapy, the 5-year survival rate of GC patients is less than 30% due to late diagnosis and tumor heterogeneity [3, 4]. The pathogenesis of GC is highly complex and controlled by genetic, epigenetic and environmental factors [4, 5]. Thus, we urgently need to find new diagnostic methods and therapeutic goals to improve clinical efficacy of the treatment for GC patients.

Evidence has suggested that circRNAs are abnormally expressed in cancer, including gastric cancer [6–9]. CircRNAs are covalently closed noncoding transcripts lacking 5′–3′ polarities with limited protein-coding potential and polyadenylated tails that are produced by the back-splicing process [10]. The closed structure of the circRNAs also renders them less susceptible to endogenous RNases, resulting in more excellent stability compared to the linear mRNAs [11, 12]. Recent reports showed that circRNAs might function as competing endogenous RNA (ceRNA), binding microRNA response elements, and have great biological significance [13–16]. Furthermore, an increasing number of researches have revealed that circRNAs play a critical role in the progression of cancers via modulating various cellular mechanisms that are essential for tumorigenesis, including cell proliferation, invasion, migration, and apoptosis [13–16]. Moreover, circRNAs could participate in carcinogenesis by modulating autophagy [17, 18].

Exosomes are endocytic vesicles measuring 30–200 nm in diameter that are secreted by cells, and aid intercellular communication between cells and their microenvironment by transporting proteins, nucleic acids, and other bioactive compounds as extracellular messengers [19, 20]. Exosomal circRNAs are circulating biomarkers of various pathological conditions, including cancer since they stable in circulation and have the diagnostic capability to distinguish patients from healthy [21], [22, 23]. Additionally, exosomal circRNAs can serve as biomarkers for liquid biopsy [24].

In the present study, we observed a novel GC-related circRNA hsa_circ_0001400, derived from exons 4, 5, and 6 through back splicing (chr4:37633006-37640126, termed circRELL1, with the length of 434 bp), which was significantly downregulated in GC tissues and plasma exosomes, while no literature was accessible to its function. With the deepening of research, circRELL1 sponges miR-637 to exert ceRNA function to regulate EPHB3 expression and autophagy activation in vivo and in vitro, which may provide a promising circulation biomarker and a novel therapeutic target in future treatments.

## Results

### CircRELL1 is downregulated in GC and associated with poor prognosis

**Fig. 1:**
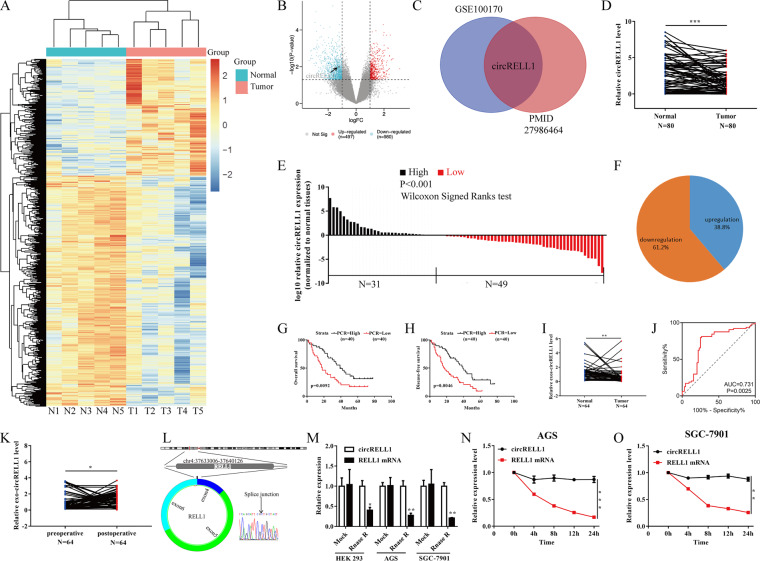
CircRELL1 is downregulated in GC and associated with poor prognosis. **A** Heat map analysis of circRNA expression profile in GC samples based on the GSE 100170. **B** Volcano plot showing circRNA transcript levels in GC and matched normal tissues. **C** The consistent circRELL1 expression between GSE 100170 and PMID27986464. **D**–**F** qRT-PCR results showing circRELL1 expression in 80 paired GC and adjacent normal gastric tissues. **G**, **H** Kaplan–Meier analysis of the correlation between circRELL1 expression and the overall survival (OS) and disease-free survival (DFS) based on circRELL1 expression levels. **I** Relative levels of plasma exosomal circRELL1 in GC patients (*n* = 64) and healthy controls (*n* = 64). **J** ROC analysis assessing the sensitivity and specificity of plasma exos-circRELL1 in predicting GC. **K** The expression of exosomal circRELL1 before and after gastrectomy (*n* = 64). **L** CircRELL1 sequence in CircBase (upper part) and validation with sanger sequencing (lower part). **M** Effect of RNase R on circRELL1 in HEK-293, AGS, and SGC-7901 cell lines. **N**, **O** Effect of actinomycin D on circRELL1 in AGS and SGC-7901 cell lines at the indicated time points. Data indicate mean ± SD. *P < 0.05, **P < 0.01, ***P < 0.001.

The differentially expressed circRNAs of 5 GC and adjacent normal tissues (GSE 100170) [25] from the GEO database were screened and observed an obviously downregulated circRNA circRELL1 (Fig. 1A, B and Supplementary Fig. s1a, b). Consistently, another microarray also demonstrated the decreased circRELL1 expression in GC (Fig. 1C). Then, circRELL1 was identified as low expression levels in 80 paired GC tissue (Fig. 1D–F). The clinicopathological features in GC tissues revealed that low circRELL1 levels were remarkably associated with poor differentiation, advanced stage, and lymph node metastasis (Supplementary Table 1 and Supplementary Fig. s1c, d). Additionally, Kaplan–Meier plot illustrated that lower circRELL1 expression was correlated with poor disease-free survival and overall survival (Fig. 1G, H). Next, after identifying plasma exosomes, depressed circRELL1 levels were tested in GC plasma exosomes compared with healthy controls with respective area under curve (AUC) values of 0.731 (Supplementary Fig. S1e and Fig. 1I, J). Furthermore, the expression of circRELL1 in plasma exosomes was consistent with GC tissues, making it possible to test the circRELL1 levels in blood samples. Besides, lower exosomal circRELL1 levels correlated with worse tumor grade, tumor stage, clinical grade, and lymphatic invasion (Supplementary Table 2). In addition, plasma exosomal circRELL1 levels were integrally increased after gastrectomy (R0 resection), indicating that exosomal circRELL1 originated from the GC tissues (Fig. 1K). The junction site of circRELL1 was examined using Sanger sequencing (Fig. 1L). Compared with RELL1 mRNA, circRELL1, as a circular form, resist to degradation by RNase R or actinomycin D (Fig. 1M–O). Moreover, the stability of plasma exosomal circRELL1 was further tested for plasma stored at room temperatures varying durations and multiple freeze-thaw cycles [26] (Supplementary Fig. S1f, g). Taken together, our results indicated that circRELL1 was downregulated in GC tissues, and could be effectively transmitted into the circulation through exosomes, and related with unfavorable prognosis, making it a potential promising circulation biomarker for GC.

### CircRELL1 transport between cells could be mediated via exosomes

**Fig. 2:**
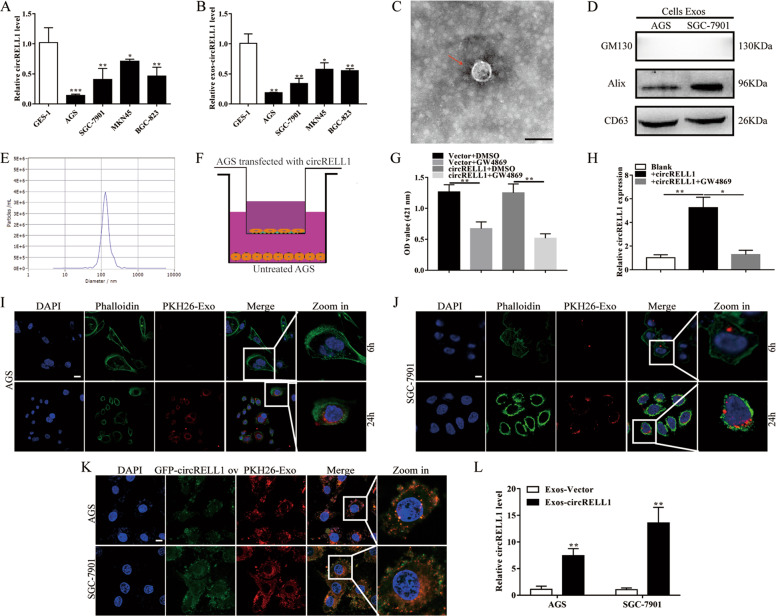
CircRELL1 transport between cells could be mediated via exosomes. **A**, **B** QRT-PCR analysis of the circRELL1 levels in the GES-1, AGS, SGC-7901, MKN45, and BGC-823 cells and their medium exosomes. **C**–**E** Transmission electron microscope (TEM), WB, and NTA of the exosomes derived from AGS medium (scale bar = 100 nm). **F** AGS cells transfected with GFP-circRELL1-overexpressing plasmid or vector were cocultured with AGS cells in a Transwell (membrane pore = 0.4 μm) plate. **G** A release of exosomes in AGS treated with GW4869 or DMSO, as Ach E activity assay determined. **H** Levels of circRELL1 in AGS treated with the medium following or not GW4869 treatment, as qRT-PCR determined. **I**, **J** Coculture of AGS and SGC-7901 with the exosomes for 6 and 24 h. Representative fluorescence images showing PKH26-labeled exosomes (red) and phalloidin-labeled F-actin (green) in the indicated cells counterstained with DAPI (blue) (scale bar: 20 μm). **K** Exosomes isolated from GC cell conditioning medium labeled with PKH26 (red) and transfected with GFP-circRELL1 (green) were cocultured with AGS and SGC-7901 cells for 24 h, while DAPI (blue) was used to stain nuclei (scale bar: 20 μm). **L** The efficiency of exosomes in delivering circRELL1 to AGS and SGC-7901 cells, as qRT-PCR analysis determined. Data indicate mean ± SD. *P < 0.05, **P < 0.01, ***P < 0.001.

Consistent with the in vivo results, decreased circRELL1 levels were detected in the 4 human GC cells lines relative to GES-1 cells (Fig. 2A). After identifying exosomes derived from AGS cell culture medium (CM), exosomal circRELL1 was also downregulated in GC cell lines relative to GES-1 cells (Fig. 2B–E). Besides, the result of extracellular circRELL1 of distribution revealed that circRELL1 was mainly located in exosomes, indicating that extracellular circRELL1 was encapsulated by exosomes instead of being directly released into the blood (Supplementary Fig. S2a). To further elucidate whether exosomal circRELL1 influenced the biological functions of GC cells, GW4869 was added to the coculture experiments and the results showed that the release of the exosomes and exosomal circRELL1 levels were regulated in the GW4869 group (Fig. 2F–H). Furthermore, the abilities of proliferation, migration, and invasion were significantly enhanced in the GW4869 group (Supplementary Fig. S2b–e). Subsequently, PKH26-labeled exosomes extracted from the cell supernatant were incubated with GC cells for 6 and 24 h, and the exosomes were gradually engulfed by receptor cells (Fig. 2I, J). To visualize the exosome-mediated intercellular circRELL1 transfer, exosomes tracing experiment demonstrated GFP-circRELL1 was co-localized with PKH26-labeled exosomes in the cytoplasm (Fig. 2K). Additionally, qRT-PCR assay illustrated that circRELL1 vector/plasmid could be packaged by exosomes and transferred into the extracellular medium, where recipient cells uptake mediated the circRELL1 expression (Fig. 2L).

### Intercellular transfer of exosomal circRELL1 suppresses the malignant phenotype in vitro

**Fig. 3:**
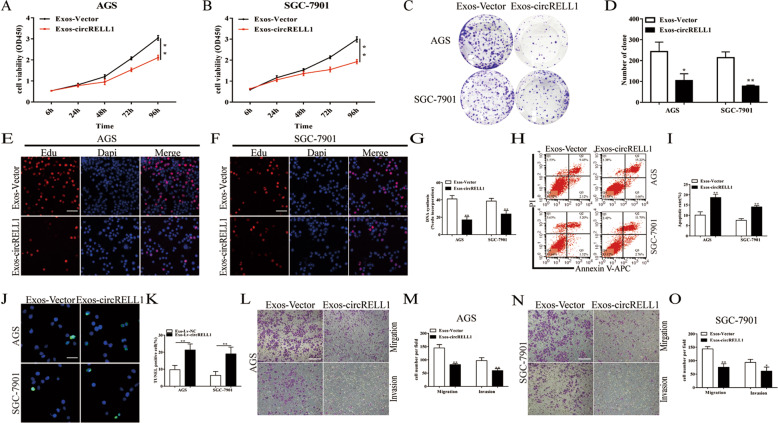
Intercellular transfer of exosomal circRELL1 suppresses the malignant phenotype in vitro. **A**, **B** CCK-8 assay was utilized to measure cell viability in GC cells after co-culturation with exos-circRELL1 or exos-vector derived from GC cells medium. **C**, **D** Colony formation assays were applied to measure cell proliferation ability in GC cells. **E**–**G** Edu assays were used to explore cell proliferation capability in GC cells (scale bar: 100 μm). **H**–**K** Flow cytometry assays and TUNEL assays were conducted to access cell apoptosis rates in GC cells (scale bar: 100 μm). **L**–**O** Transwell assays were employed to investigate cell migration and invasion capability in GC cells (scale bar: 200 μm). Results are presented as mean ± SD of three independent experiments. *P < 0.05, **P < 0.01.

Exosomes are known to mediate intercellular communication and may change the physiological function of the receptor cells through biologically active factors, including circRNAs [27, 28]. For further investigating exosomal circRELL1 function in the GC cells, exosomes from the CM of GC cells transfected with circRELL1-overexpressing plasmid or NC vector were extracted. CCK-8, colony formation, Edu, Flow cytometric, TUNEL, and transwell assays were utilized and we observed that strengthening exosomal circRELL1 could significantly weaken cell proliferation ability, anti-apoptosis, and migration ability in comparison with the negative control, blocking malignant cell phenotypes (Fig. 3A–O). Thus, more circRELL1 was packaged into exosomes with the ectopic circRELL1 expression in GC cells, disturbing the biological behavior of adjacent or distant GC.

### Exomsomal circRELL1 decreases GC organoids proliferation and inhibits tumor growth and metastasis in vivo

**Fig. 4:**
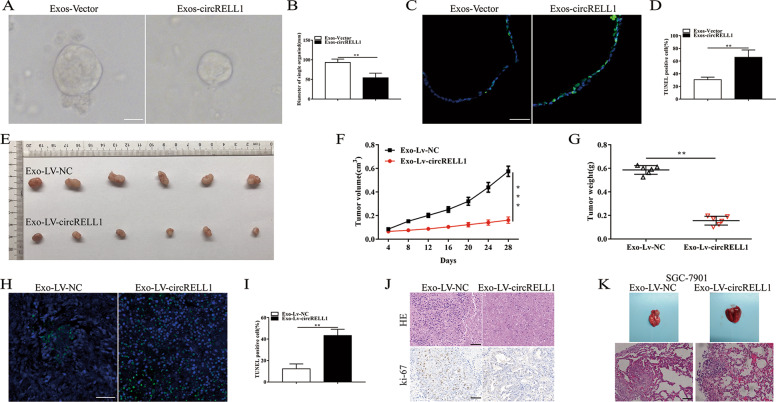
Exomsomal circRELL1 decreases GC organoids proliferation and inhibits tumor growth, and metastasis in vivo. **A**, **B** Representative results of human GC organoids culture after co-culturation with circRELL1-overexpressing exosomes or control exosomes (scale bar: 25 μm). **C**, **D** Assessment of the apoptosis of organoids cocultured with exos-circRELL1 or exos-Vector by TUNEL assays. **E** SGC-7901 transfected with Exo-LV-circRELL1 or Exo-LV-NC was inoculated in the burden nude mice. **F**, **G** Analysis of tumor size and weight after different treatments. **H**, **I** Assessment of the apoptosis of the xenografts by TUNEL assays (scale bar: 50 μm). **J** Representative results of HE staining of the specimen and assessment of the Ki-67 protein levels in the xenografts by IHC analysis (scale bar: 50 μm). **K** Representative images of pulmonary metastasis and HE staining of specimen (scale bar: 50 μm). Results are presented as mean ± SD of three independent experiments. **P < 0.01, ***P < 0.001.

To further evaluate the exosomal circRELL1 biological functions in tumor growth, human GC organoids were established and the results suggested that exosomal circRELL1 remarkablely repressed the proliferation capacity and facilitated apoptosis capacity in terms of diameter and TUNEL of the organoids (Fig. 4A–D). To investigate whether exosomal circRELL1 affected tumor growth in vivo, SGC-7901 cells were injected subcutaneously into nude mice, following SGC-7901 cell-derived Exo-Lv-NC and Exo-Lv-circRELL1 preincubation. The results illustrated that the smaller tumors were formed in the Exo-Lv-circRELL1 group (Fig. 4E–G). Furthermore, TUNEL assays revealed an increase in apoptotic rate after treatment with Exo-Lv-circRELL1 (Fig. 4H, I). Moreover, HE and IHC staining indicated that tumor tissue from nude mice ki-67 expression was lower in the Lv-circRELL1 group than the control group (Fig. 4J). To probe the role of exosomal circRELL1 on tumor metastasis in vivo, the lung metastasis model was established and we found that exosomal circRELL1 alleviated the lung metastasis (Fig. 4K). Thence, the aforementioned results showed that circRELL1 induced apoptosis and inhibited the growth of the GC organoids, suppressed GC tumor proliferation, and metastasis in vivo.

### CircRELL1 suppresses the malignant transformation of GC in vitro and in vivo

**Fig. 5:**
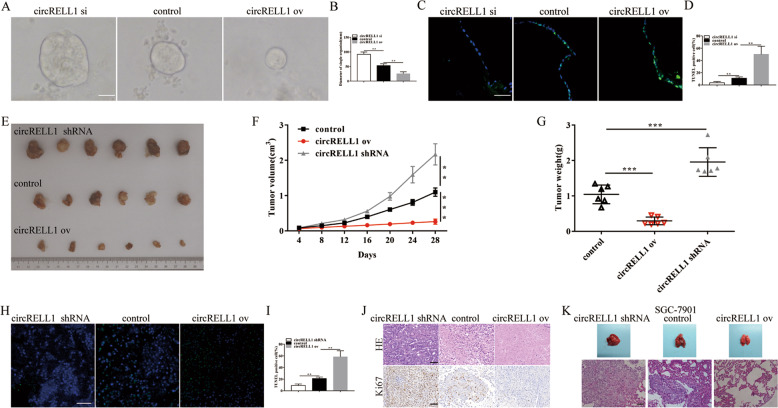
Enhanced circRELL1 suppresses GC organoids proliferation and represses tumor growth, and metastasis in vivo. **A**, **B** Representative results of human GC organoids culture after circRELL1-overexpressing plasmid, circRELL1 shRNA, or control transfection (scale bar: 25 μm). **C**, **D** Assessment of the apoptosis of organoids cocultured with different treatments by TUNEL assays. **E** Xenograft tumors under different treatments. **F**, **G** Analysis of tumor size and weight after different treatments. **H**, **I** Assessment of the apoptosis of the xenografts by TUNEL assays (scale bar: 50 μm). **J** Representative results of HE staining of the specimen and assessment of the Ki-67 protein levels in the xenografts by IHC analysis (scale bar: 50 μm). **K** Representative images of pulmonary metastasis and HE staining of specimen (scale bar: 50 μm). Results are presented as mean ± SD of three independent experiments. **P < 0.01, ***P < 0.001.

To validate the biological role of circRELL1 in GC cells, two siRNAs were designed to target the junction site and then, transfection of RNA interference and overexpression plasmids were applied for the following experiments (Supplementary Fig. S3a–d). CCK-8 and EdU assays revealed that strengthening circRELL1 significantly impaired cell proliferation capability in comparison with the control, while silencing circRELL1 could increase GC cells proliferation (Supplementary Fig. S4a–j). Next, Flow cytometric assays indicated that enhanced circRELL1 advanced the apoptosis, while decreased circRELL1 dramatically attenuate the apoptosis of both GC cells (Supplementary Fig. S4k–p). Furthermore, human GC organoids were built to investigate the proliferation of tumors and the results indicated that the strengthened circRELL1 blocked the proliferation and anti-apoptosis abilities of organoids, while weaken circRELL1 had the opposite effect (Fig. 5A–D). Subsequently, SGC-7901 cells were stably transfected with lentiviral circRELL1-overexpressing, shRNA targeting circRELL1 or control, which were inoculated subcutaneously into nude mice to evaluate the effect of circRELL1 on tumor growth in vivo, and we observed that enhanced circRELL1 markedly decreased tumor volume and weight, while the tumor growth was promoted after knockdown of circRELL1 (Fig. 5E–G). Additionally, tumor tissues were applied to examine the abilities of anti-apoptosis and proliferation by HE, ki-67 IHC, and TUNEL assays, it demonstrated that weaken circRELL1 facilitated anti-apoptosis and proliferation of the xenografts, while strengthened circRELL1 had the opposite effect (Fig. 5H–J). To investigate the role of circRELL1 on tumor metastasis in vivo, stable cells treated differently were injected into nude mice via tail vein and the results revealed that lung metastasis in circRELL1 overexpression group was alleviated, while weaken circRELL1 promoted lung metastasis (Fig. 5K). Thus, enhancing circRELL1 repressed GC tumor growth and metastasis.

### CircRELL1 acts as a miRNA sponge for miR-637

**Fig. 6:**
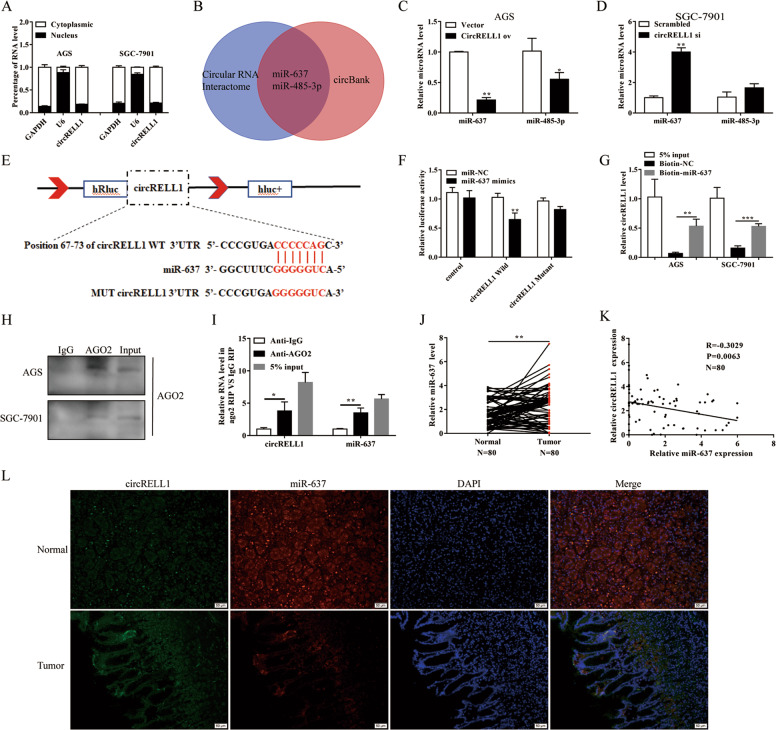
CircRELL1 acts as a miRNA sponge for miR-637. **A** qRT-PCR analysis was applied to measure subcellular localization of circRELL1 in GC cells. **B** Target miRNAs of circRELL1 were predicted by Circular RNA Interactome and circBank. **C**, **D** The relative miRNAs levels in the AGS and SGC-7901 cells after different treatments. **E** The predicted binding sites between circRELL1 and miR-637. **F** Dual-luciferase assay was performed to investigate the direct binding between circRELL1 and miR-637 in HEK-293 cells. **G** CircRELL1 were pulled down by biotin-labeled miR-637 and confirmed by qRT-PCR in AGS cells. **H**, **I** The assessment of the enrichment in Ago2 immunoprecipitates, as determined by WB and qRT-PCR. **J** qRT-PCR analysis of miR-637 levels in the paired GC and adjacent normal tissues. **K** Correlation analysis of the relationship between circRELL1 and miR-637 in 80 GC tissues. **L** FISH analysis of circRELL1 and miR-637 levels in GC and adjacent tissues. Results are presented as mean ± SD of three independent experiments. **P < 0.01, ***P < 0.001.

To further examine how circRELL1 exerts its function, the cytoplasmic location of circRELL1 indicated that circRELL1 may regulate the progression of GC at the posttranscriptional level (Fig. 6A). Hereafter, candidate miRNAs that may bind with circRELL1 were predicted using Circular RNA Interactome and circBank (Fig. 6B). The downstream miRNAs were detected after gain or loss of circRELL1 and the results indicated that miR-637 was the significant changed one (Fig. 6C, D). Dual-luciferase reporter assays confirmed that miR-637 could bind directly to the circRELL1 3′UTR (Fig. 6E, F). Next, pull-down assays revealed that circRELL1 was significantly enriched using biotin-labeled miR-637 probe compared with the control group (Fig. 6G). Furthermore, RIP assay demonstrated that circRELL1 and miR-637 were recruited by Ago2 (Fig. 6H, I and Supplementary Fig. S5a). Additionally, we verified the higher expression of miR-637 among 80 pairs GC tissues and observed a significant inverse correlation between circRELL1 and miR-637 levels (Fig. 6J, K). FISH of GC tissues showed the co-localization and opposite expression between circRELL1 and miR-637 (Fig. 6L). Subsequently, clinical data illustrated that the high miR-637 levels were positively correlated with poor T stages and TNM stages (Supplementary Fig. S5b, c). Kaplan–Meier Plotter elucidated that patients with high miR-637 levels had lower overall survival (OS) and disease-free survival (DFS) (Supplementary Fig. S5d, e). Collectively, these results proved that circRELL1 directly “sponges” miR-637.

### The negative regulation of circRELL1 is partially mediated by miR-637

**Fig. 7:**
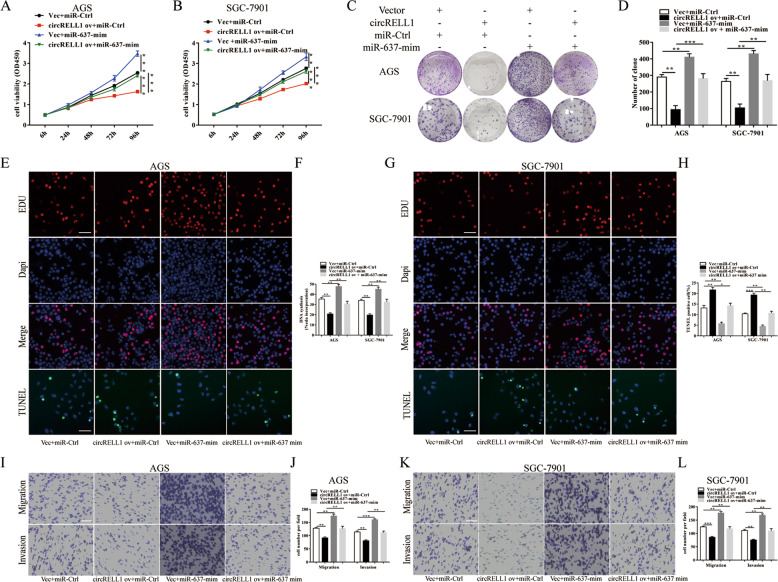
The negative regulation of circRELL1 is partially mediated by miR-637. **A**–**H** Results of CCK-8, colony formation, TUNEL assays, and Edu assays were applied to investigate the ability of proliferation after co-transfection with circRELL1-overexpressing plasmid, miR-637 mimic, or controls (scale bar: 100 μm). **I**–**L** The transwell assays were conducted to explore the cell migration and invasion capability under different treatments (scale bar: 100 μm). All data were presented as the mean ± SD. *P < 0.05, **P < 0.01, ***P < 0.001.

After verifying the transfection efficiency of miR-637 (Supplementary Fig. S5f), AGS and SGC-7901 cells were co-transfected with circRELL1 plasmid and miR-637 mimics to investigate the potential mechanisms of circRELL1/miR-637 regulation of GC progression. Subsequently, CCK-8, colony formation, Edu, TUNEL, and transwell assays were performed and the results demonstrated that enhanced circRELL1-mediated suppression of the cell proliferation, anti-apoptosis, and migration were partially reversed via co-transfection with miR-637 mimics (Fig. 7A–L). In summary, the results revealed that the anti-oncogenic role of circRELL1 was fractionally mediated by negative regulation of miR-637.

### EPHB3 as a miR-637 downstream target gene is regulated by circRELL1

**Fig. 8:**
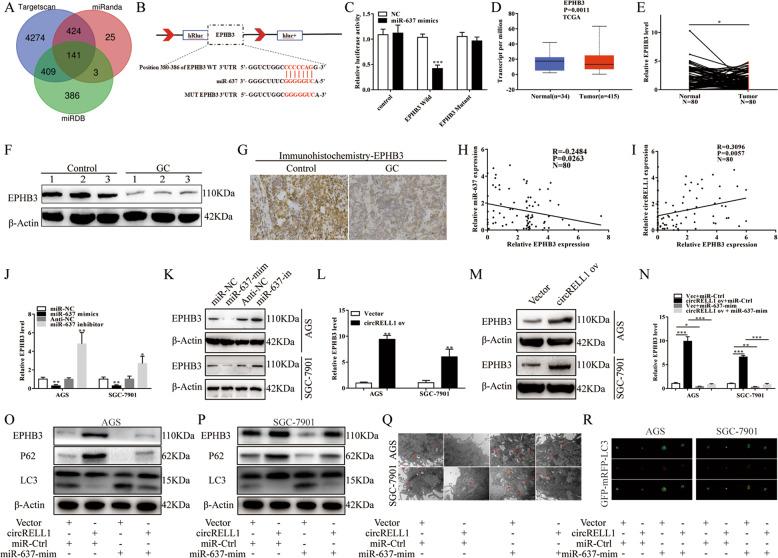
EPHB3 as a miR-637 downstream target gene is regulated by circRELL1. **A** MiR-637 targets predicted using the TargetScan, miRDB, and miRanda. **B** The predicted binding sites between miR-637 and EPHB3. **C** Dual-luciferase assay was applied to investigate the direct binding between EPHB3 and miR-637 in HEK-293 cells. **D** Relative expression of EPHB3 GC tissues in TCGA database. **E** qRT-PCR analysis of EPHB3 levels in the paired GC and adjacent normal tissues. **F**, **G** IHC and WB assays were used to measure the EPHB3 protein levels in GC samples. **H**, **I** Correlation analysis of the relationship between circRELL1, miR-637, and EPHB3 in 80 GC tissues. **J**, **K** qRT-PCR and WB analyses were applied to detect the EPHB3 levels after co-transfection with miR-637 inhibitor, miR-637 mimics, or their controls. **L**, **M** qRT-PCR and WB analyses were applied to detect the EPHB3 levels after transfection with circRELL1-overexpressing plasmid or Vector. **N**–**R** Assessment of EPHB3 levels and autophagy activation after co-transfection with circRELL1-overexpressing plasmid, miR-637 mimics, or controls by qRT-PCR, WB analyses, TEM, and confocal microscopy. Results are presented as mean ± SD of three independent experiments. *P < 0.05, **P < 0.01, ***P < 0.001.

With three bioinformatics websites: miRanda, miRDB, and TargetScan, EPHB3 was putated as a target of miR-637 after functional analysis based on the NCBI database and the expressions of the potential target genes (Fig. 8A and Supplementary Fig. S6a, b). Furthermore, dual-luciferase reporter assays illustrated that miR-637 could bind directly to the EPHB3 3′UTR (Fig. 8B, C). Besides, EPHB3 was downregulated based on the TCGA database, qRT-PCR, WB, and IHC (Fig. 8D–G and Supplementary Fig. S6c). Subsequently, EPHB3 levels were negatively related to miR-637 while positive to circRELL1, consistent with the in vivo results (Fig. 8H, I and Supplementary Fig. S6d–f). To explore whether miR-637 could regulate EPHB3, we observed that the EPHB3 levels changed with gain or loss of miR-637, respectively (Fig. 8J, K and Supplementary Fig. S6g). To investigate whether circRELL1 can regulate EPHB3, the results illustrated that enhanced circRELL1 still increased the EPHB3 mRNA and protein levels (Fig. 8L–M and Supplementary Fig. S6h–j). Studies have shown that autophagy could satisfy the high metabolic demands for cancer survival and proliferation [29] [30]. WB analysis showed that the LC3 level was decreased in cells treated with exos-circRELL1 3-MA, and increased in rapamycin-treated cell (Supplementary Fig. S7a, b). Next, we found that EPHB3 levels and autophagy activity medicated by circRELL1 overexpression were partially counteracted by miR-637 mimics in the rescue experiment (Fig. 8N–R and Supplementary Fig. S7c–e). Kaplan–Meier Plotter elucidated that patients with high EPHB3 levels had higher PFS and OS based on the TCGA database (Supplementary Fig. S7f–h). After accessing the transfection efficiency of EPHB3 (Supplementary Fig. S7i), AGS and SGC-7901 cells were co-transfected with EPHB3 plasmid and miR-637 mimics to verify the interactions between EPHB3 and miR-637 in GC. Subsequently, CCK-8, colony formation, Edu, TUNEL, and transwell assays revealed that enhanced EPHB3-mediated suppression of the cell proliferation and migration were partially reversed by co-transfection with miR-637 mimics (Supplementary Fig. S8a–l). Taken together, these data showed that circRELL1 primarily modulated the expression of EPHB3 and autophagy activity by miR-637 at the posttranscriptional level.

## Discussion

Accumulated studies show that dysregulated circRNAs play a critical role in malignant development and tumor growth, including GC [31, 32]. However, the molecular mechanisms underlying the functional role of many GC associated circRNAs have not yet been explored in depth. In the current effort, we first identified a GC-related circRNA from the RELL1 gene, circRELL1, which has not been reported in other tumors and was reduced in GC tissues and cells. CircRELL1 derived from plasma exosomes was similarly expressed in the plasma exosomes of GC patients. The enhanced exosomal circRELL1 after gastrectomy involved that plasma exosomal circRELL1 originated from GC tumors and exosomal circRELL1 might serve as a liquid biopsy for GC. In addition, the decreased expression of circRELL1 was related to advanced TNM stage and poor prognosis. Further analysis demonstrated that enhanced circRELL1 and exosomal circRELL1 weakened cell proliferation, anti-apoptosis, tumor growth, and migration in vitro and in vivo, while knockdown of circRELL1 advanced cell proliferation and anti-apoptosis. These findings illustrated that circRELL1 exerted a vital role as a suppressor in gastric tumorigenesis and may function as a stable circulation biomarker for the diagnosis and prognosis in GC.

Recently, much attention was focused on the ceRNA hypothesis and circRNA may sponge corresponding miRNAs to modulate the target genes of the miRNAs [15, 33, 34]. In this study, subcellular localization of circRELL1 was examined and we found that majority of circRELL1 was located in cytoplasm, indicating that circRELL1 may serve as a ceRNA for sponging miRNAs. Furthermore, bioinformatics analyses, RNA pull-down, RIP assays, and luciferase reporter assays indicated that miR-637 was a new target of circRELL1. We found that miR-637 was obviously overexpressed in GC samples, and its expression was inversely correlated with circRELL1. Furthermore, rescue assays indicated that overexpression of miR-637 could partially reverse the effects of GC cell function mediated by enhanced circRELL1. Intracellularly, circRELL1 plays a crucial role in GC cells via sponging miR-637. Dysfunctional circRELL1 transferred by exosomes could be transported into peripheral circulation, regulating neighboring or distant GC cell growth and metastasis.

Generally, circRNAs act as a ceRNA, exerting their function by regulating miRNA targets. Bioinformatics analyses and luciferase reporter assays showed that EPHB3 was a new target of miR-637. EPHB3, a receptor tyrosine kinase (RTK) with varying carcinogenic effects, has been reported to modulate the progression of various tumors [35], [36], [37]. Further experiments revealed that EPHB3 expression was downregulated in GC samples and the correlation between circRELL1, miR-637, and EPHB3 expression was also examined both in vivo and in vitro. Besides, rescue experiments demonstrated that strengthening EPHB3 could partly reverse proliferation, migration, and invasion capabilities of tumor cells induced by miR-637 upregulation and exhibit regulative effects on GC. Hence, these results indicated that circRELL1 sponged miR-637 and knockdown the expression of its direct endogenous target EPHB3 in GC.

Accumulated evidence suggests that cancers can use autophagy to support their elevated metabolic demand for growth and proliferation [29]. For instance, Alicia et al. verified that oncogenic autophagy occurs in prostate cancer [38]. Xu et al. found that autophagy activation served as a protective mechanism in GC [39]. In addition, researches have revealed that autophagy was regulated by circRNAs [40]. CircDNMT1 interacted with p53 and AUF1 to activate autophagy in breast cancer [41]. CircHIPK3 regulated autophagy activation by sponging miR-124 in lung cancer [42]. Moreover, previous studies demonstrated that circRNA regulated downstream target genes through the mechanism of ceRNA-mediated autophagy and mediated malignant phenotype in GC [43]. The ubiquitination of EPHB2 participated in macroautophagy/autophagy activation in the DSS-induced colitis model [44]. Intriguingly, the findings of this study indicated that circRELL1/miR-637/EPHB3 regulated autophagy activation to mediate the biological functions in GC.

However, our study also has some limitations. Firstly, we cannot exclude the role of circRELL1 in suppressing GC through other pathways due to the complex interaction between cytokines. Moreover, this signaling axis is limited to AGS and SGC-7901 cell lines and further studies should be performed to explore other GC cell lines.

To sum up, we discovered a novel GC-related circRNA circRELL1 and decreased circRELL1 was associated with advanced TNM stage and poor prognosis. Enhanced circRELL1 promoted EPHB3 to weaken GC proliferation, autophagy, migration, and invasion through sponging of miR-637 in vitro and in vivo. Besides, plasma exosomal circRELL1 could be helpful for diagnosis, prediction, and progression (Supplementary Fig. S9). Consequently, circRELL1 is a promising stable diagnostic biomarker and therapeutic target in GC.

## Materials and methods

### Patient samples

Raw RNA microarray data were downloaded from the GEO database (https://www.ncbi.nlm.nih.gov/gds/) and were analyzed by R software (version 3.3.4). The UCSC cancer browser with version number 07-20-2019 (https://xenabrowser.net/datapages/) offered the TCGA gene datasets including TCGA-STAD.htseq_fpkm-uq.tsv (involving 372 GC tissues and 35 normal tissues) and TCGA-STAD.mirna.tsv (involving 436 GC tissues and 41 normal tissues). A total of 80 pairs of GC organizations and adjacent non-cancer tissues were obtained from patients who underwent surgery at First Affiliated Hospital of Nanjing Medical University. The histological grading was based on the eighth tumor-lymph node metastasis (TNM) staging of the American Joint Committee on Cancer. Sixty-four cases with GC and 64 healthy controls that provided human plasma samples were matched according to gender, age, and disease. This research was approved by the Clinical Research Ethics Committee of The First Affiliated Hospital of Nanjing Medical University, moreover written informed consent was acquired from all participants.

### Cell culture

Four human gastric cancer cell lines (AGS, SGC-7901, MKN45, and MGC823) and two normal cell lines (GES-1 and HEK-293) were purchased from the Cell Bank of Type Culture Collection of the Chinese Academy of Sciences, maintained under 37 °C, 5% CO_2_ in RPMI-1640 medium assisted with 10% fetal bovine serum (FBS, Gibco-BRL), 100 U/mL penicillin and 100 mg/mL streptomycin.

### Isolation and identification of exosomes

The cells were cultured with FBS media without exosomes. When the cells attained confluency of 70–80%, they were washed three times with PBS and changed to serum-free media for 24 h, and then the supernatant was collected. After that, follow the standard procedure described previously to extract exosomes from the culture media by ultracentrifugation [45]. For exosomes of plasma, exoEasy Maxi Kit (Qiagen, Hilden, Germany; Cat. Number:76064) was conducted to isolate exosome RNA according to the protocol of the manufacturer. The exosomes were eventually separated and assigned to RNase-free tubes for storage or electron microscopy(FEI Company, USA), protein assays, and to be used for intervention.

### Exosome labeling and uptake

Exosomes were labeled with PKH26 red fluorescent dye (Sigma-Aldrich, USA) following the manufacturer’s protocol. A total of 50 μg of exosomes were resuspended in 100 μl PBS and were added to 5 × 10^5^ gastric tumor cells. Faction was stained with phalloidin-FITC (green), and DAPI (blue) was used to keep the nucleus. To track exosomes, exosomes secreted from GC cells were labeled with PKH26 red fluorescent dye (Sigma-Aldrich, USA), while circRELL1 was marked with GFP (green), and DAPI (blue) was used to mark the nucleus. Subsequently, a fluorescence microscope (Zeiss, LSM700B, Germany) was adopted to examine stained cells. The uptake capacity of AGS and SGC-7901 into exosomes (exos-Vector/exos-circRELL1) was assessed via immunofluorescence assays and qRT-PCR.

### Transfection

Cells were transfected using Lipofectamine 3000 (Invitrogen, Carlsbad, CA, USA) according to the instructions for transfection reagents. RELL1 overexpression plasmid, small interfering RNA, empty vector (NC), or microRNA-637 (miRNA-637) mimics inhibitors were synthesized by GenePharma (Shanghai, China). The lentiviral vectors overexpressing circRELL1/NC were purchased from GeneChem (Shanghai, China).

### RNA extraction and quantitative qRT-PCR assays

Total RNA from tissues and cells lines was extracted using TRIzol reagent (Invitrogen, USA) and NanoDrop 2000 spectrophotometer (Thermo Scientific, USA) was conducted to accomplish RNA quantity control and concentration detection. RNase R (Epicentre Biotechnologies, USA) experiment (15U) was applied on total RNA (5 ug) for 15 min at 37 °C. Transcription Kit (TaKaRa, RR036A, Japan) was then utilized to reverse the RNAs into cDNA and subsequently applied qRT-PCR on the LightCycler480II (Switzerland) to estimate the expression of mRNA or miRNA. MRNA results were normalized to the expression of GAPDH, while miRNA normalized to U6, and both were calculated to fold changes using 2(−ΔΔCT). The primers involved in this study were listed in Supplementary Table 3.

### Protein extraction and western blotting (WB)

RIPA buffer (Sigma-Aldrich, USA) mixed with a protease inhibitor cocktail (Roche) were adopted to extract total proteins from samples and protein concentrations were examined by the bicinchoninic acid (BCA) solution (Pierce, USA). SDS-containing polyacrylamide gel (SDS-PAGE) was conducted to separate cell protein lysates, which were transmitted to 0.22 mm polyvinylidene fluoride membranes (PVDF) membrane (Bio-Rad, USA) and investigated with specific antibodies. EPHB3 (PA5-82337) were purchased from Invitrogen (USA); GM130 (ab52649), p62 (ab56416), CD63 (ab217345), and Alix (ab88388) were purchased from Abcam (USA); LC3 (#12741) was purchased from Cell Signaling Technology (USA); Beta Actin (20536-1-AP) was purchased from Proteintech (China). The standard WB was conducted under the primary antibodies and secondary antibodies.

### Cell counting kit-8 assay

The proliferation activities of GC cells were accessed by a Cell Counting Kit-8 assay (Dojindo Laboratories, Kumamoto, Japan). Cells were seeded into 96-well plates and incubated with 10 μL of CCK-8 solution per well for 1 h on 5 days. The absorbance of the test wells was measured for analysis. A microplate reader (ELX808; Bio Tek, Winooski, VT, USA) was adopted to test the absorbance of the wells at 450 nm.

### 5-Ethynyl-2′-deoxyuridine (Edu) assay

The proliferation capacity of the GC cells was evaluated by an Edu assay kit (RiboBio, China). In brief, the treated cells were stained with Edu and DAPI following the standard instructions of the manufacturer, and then fluorescence microscope (Nikon, Japan) was applied to observe the cells.

### Colony formation

The treated GC cells were planted and incubated in six-well plates at 37 °C for 2 weeks. After formaldehyde fixation and crystal violet staining, the cell colonies were imaged and counted to measure the proliferation activities.

### Transwell assay

The GC cells were planted into the upper chambers with or without Matrigel (BD Biosciences, USA) in serum-free medium, while the lower chamber maintained medium with 10% FBS at 37 °C for 24 h. After fixation and staining, a microscope was used to image and count the cells.

### Apoptosis assay

The treated cells were stained via Apoptosis Detection Kit (BD Biosciences #556547) following the instructions and all apoptotic cells were calculated to measure the apoptotic rate.

### Fluorescence in situ hybridization (FISH) and immunohistochemistry (IHC)

Specific probes targeting circRELL1 and miR-637 sequence were synthesized by RiboBio (Guangzhou, China) and GenePharma (Shanghai, China), used to perform FISH as described in a previous research [46]. IHC staining was performed as described previously [47].

### Dual-luciferase reporter assay

The 3′ UTR sequences of circRELL1 and EPHB3 were cloned into a pGL3 promoter (Genechem, Shanghai, China). The reporter plasmid, miR-637 mimics and negative control were transfected into cells using lipofectamine 3000. The Dual-Luciferase Assay (Promega, Madison, WI, USA) was utilized to investigate the luciferase activities in the treated Cells.

### RNA immunoprecipitation (RIP)

An RNA-Binding Protein Immunoprecipitation Kit (17–700, Merck, Millipore) was purchased to perform a RIP assay. Briefly, magnetic beads were incubated with Argonaute2 (Ago2; Millipore), or IgG and then were added into the cell lysates. The measure conformed to the instructions of the manufacturer supplier. Immunoprecipitated RNA was ultimately detected by qRT-PCR and WB to test the presence of circRELL1 using specific primers.

### RNA pull-down

In briefly, prewashed streptavidin beads were incubated with bio-miR-637 probe and then were added into the cell lysates. After added with wash buffer and proteinase K, the hybridized RNA was measured by qRT-PCR.

### GC organoids

In brief, glands were extracted from 1 cm^2^ of human GC tissue using EDTA in cold chelation buffer, seeded in Matrigel (BD Biosciences) and overlaid with the medium as described to simulate the microenvironment of GC [48]. Exos-circRELL1 and circRELL1 plasmid were transfected into the organoids to access the exos-circRELL1 in GC progression. Human GC organoid growth was observed daily by microscope.

### Animal experiments

For the tumor xenograft model, a total of 30 mice were randomly divided into five groups, and differently treated cells were injected subcutaneously into 6-week-old female BALB/c nude mice. The tumor weight was monitored and volume was counted every 4 days. For the lung metastasis model, the SGC-7901 cells were injected into the caudal vein of nude mice (four mice/group). Nanjing Medical University’s Institutional Animal Care and Research Advisory Committee Approval were obtained to conduct the animal experiments.

### Statistical analysis

Chi-square tests and Student’s *t*-test were used to access statistical differences between GC samples by GraphPad Prism (version 7.0). All results from experiments were independently performed in triplicate, presented in the form of mean ± SD. The log-rank test and Kaplan–Meier analysis constituted the survival analysis. A statistically significant result was indicated by a *P* value of <0.05.

## Supplementary information

Supplementary Table 1

Supplementary Table 2

Supplementary Table 3

Supplemental Figure Legends

Supplementary Figure 1

Supplementary Figure 2

Supplementary Figure 3

Supplementary Figure 4

Supplementary Figure 5

Supplementary Figure 6

Supplementary Figure 7

Supplementary Figure 8

Supplementary Figure 9

## Data Availability

The data that support the findings of this study are available on request from the corresponding author.
