# Supplementary material for: Exosomal circRELL1 serves as a miR-637 sponge to modulate gastric cancer progression via regulating autophagy activation
Source: Cell Death Dis. 2022 Jan 13;13(1):56. doi: 10.1038/s41419-021-04364-6 (PMC8758736; doi:10.1038/s41419-021-04364-6)
Supplement: Supplementary file 1 — Supplementary Table 1 [file 41419_2021_4364_MOESM1_ESM.docx]

**Supplementary Table 1.** **Clinical parameters of 80 paired tissues for circRELL1**

| Characteristics | Group | N | High expression | Low expression | P-value |  |
| --- | --- | --- | --- | --- | --- | --- |
| Gender | Female | 30 | 13 | 17 | 0.356 |  |
|  | Male | 50 | 27 | 23 |  |  |
| Age(y) | ≤60 | 28 | 13 | 15 | 0.639 |  |
|  | ＞60 | 52 | 27 | 25 |  |  |
| Histological type | Well differentiated | 43 | 26 | 17 | 0.044* |  |
|  | Poorly differentiated | 37 | 14 | 23 |  |  |
| TNM stage | I-II | 38 | 26 | 12 | 0.002** |  |
|  | III-IV | 42 | 14 | 28 |  |  |
| T stage | T1-T2 | 24 | 17 | 7 | 0.015* |  |
|  | T3-T4 | 56 | 23 | 33 |  |  |
| Lymphatic invasion | No | 31 | 20 | 11 | 0.0389* |  |
|  | Yes | 49 | 20 | 29 |  |  |

*p < 0.05, **p < 0.01.
