# Supplementary material for: Exosomal circRELL1 serves as a miR-637 sponge to modulate gastric cancer progression via regulating autophagy activation
Source: Cell Death Dis. 2022 Jan 13;13(1):56. doi: 10.1038/s41419-021-04364-6 (PMC8758736; doi:10.1038/s41419-021-04364-6)
Supplement: Supplementary file 2 — Supplementary Table 2 [file 41419_2021_4364_MOESM2_ESM.docx]

**Supplementary Table 2. Clinical parameters of 64 GC cases for plasma exo-circRELL1**

| Characteristics | Group | N | High expression | Low expression | P-value |  |
| --- | --- | --- | --- | --- | --- | --- |
| Gender | Female | 12 | 5 | 7 | 0.522 |  |
|  | Male | 52 | 27 | 25 |  |  |
| Age(y) | ≤60 | 26 | 15 | 11 | 0.309 |  |
|  | ＞60 | 38 | 17 | 21 |  |  |
| Histological type | Well differentiated | 36 | 22 | 14 | 0.044* |  |
|  | Poorly differentiated | 28 | 10 | 18 |  |  |
| TNM stage | I-II | 31 | 20 | 11 | 0.024* |  |
|  | III-IV | 33 | 12 | 21 |  |  |
| T stage | T1-T2 | 18 | 13 | 5 | 0.026* |  |
|  | T3-T4 | 46 | 19 | 27 |  |  |
| Lymphatic invasion | No | 24 | 16 | 8 | 0.039* |  |
|  | Yes | 40 | 16 | 24 |  |  |

*p < 0.05, **p < 0.01.
