# Supplementary material for: Exosomal circRELL1 serves as a miR-637 sponge to modulate gastric cancer progression via regulating autophagy activation
Source: Cell Death Dis. 2022 Jan 13;13(1):56. doi: 10.1038/s41419-021-04364-6 (PMC8758736; doi:10.1038/s41419-021-04364-6)
Supplement: Supplementary file 3 — Supplementary Table 3 [file 41419_2021_4364_MOESM3_ESM.docx]

**Supplementary Table 3. List of gene primers**

| GAPDH | Forward: GCACCGTCAAGGCTGAGAAC |
| --- | --- |
|  | Reverse: GGATCTCGCTCCTGGAAGATG |
| U6 | Forward: CTCGCTTCGGCAGCACA |
|  | Reverse: AACGCTTCACGAATTTGCGT |
| circRELL1 | Forward: GAACGGAGAAGCCTGATGTC |
|  | Reverse: GGGGCTTTCAGGATCATACA |
| linear RELL1 | Forward: GTGCTTGCTGCTTCAGTGTTCGT |
|  | Reverse: CTTCCTGTTCCGCCTCTGTTGTA |
| miR-637 | Forward: ACACTCCAGCTGGGACTGGGGGCTTTCGGGCT |
|  | Reverse: CTCAACTGGTGTCGTGGAGTCGGCAATTCAGTTGAGACGCAGAG |
| miR-485-3p | Forward: ACACTCCAGCTGGGGTCATACACGGCTCTC |
|  | Reverse: CTCAACTGGTGTCGTGGAGTCGGCAATTCAGTTGAGAGAGAGGA |
| EPHB3 | Forward: TCGTGGTCATCGCTATCGTCT |
|  | Reverse: AAACTCCCGAACAGCCTCATT |
| circRELL1 si1 | Sense: AGUAGCAGCGAAUGCUGAUTT |
|  | Antisense: AUCAGCAUUCGCUGCUACUTT |
| circRELL1 si2 | Sense: GCACAGAGUAGCAGCGAAUTT |
|  | Antisense: AUUCGCUGCUACUCUGUGCTT |
| miR-637 mimics | Sense: ACUGGGGGCUUUCGGGCUCUGCGU |
|  | Antisense: GCAGAGCCCGAAAGCCCCCAGUUU |
| miR-637 inhibitor | Sense: ACGCAGAGCCCGAAAGCCCCCAGU |
|  |  |
| **Probes** |  |
|  |  |
| Bio-miR-637 | ACGCAGAGCCCGAAAGCCCCCAGT |
| Cy3-miR-637 | ACGCAGAGCCCGAAAGCCCCCAGT |
