# Supplementary material for: Exosomal circRELL1 serves as a miR-637 sponge to modulate gastric cancer progression via regulating autophagy activation
Source: Cell Death Dis. 2022 Jan 13;13(1):56. doi: 10.1038/s41419-021-04364-6 (PMC8758736; doi:10.1038/s41419-021-04364-6)
Supplement: Supplementary file 4 — Supplemental Figure Legends [file 41419_2021_4364_MOESM4_ESM.docx]

Supplementary Figure 1.

**a, b**. GO and KEGG analyses of the host genes based on the differentially expressed circRNAs. **c, d.** The correlation between circRELL1 levels and T stage and TNM stage in 80 GC samples via qRT-PCR. **e.** Identification of the exosomes from the plasma by WB. **f.** Exos-circRELL1 expression in plasma samples after 0, 2, 4 and 8 freeze-thaw cycles. **g.** Exos-circRELL1 expression in plasma stored at room temperature for 0, 4, 8 and 24 h. Results are presented as mean ± SD of three independent experiments. *P < 0.05, **P < 0.01.

Supplementary Figure 2.

GW4869 reduces the delivered exosomal circRELL1. **a.** CircRELL1 levels in the whole plasma, supernatant and plasma exosomes, as qRT-PCR determined. **b, c.** Transwell assays were conducted to measure the migration and invcasion of AGS treated with the circRELL1-overexpressing plamid medium following or without GW4869 treatment. **d, e.** Assessment of the proliferation of the AGS under different treatments (scale bar: 100 μm). Results are presented as mean ± SD of three independent experiments. *P < 0.05, **P < 0.01.

Supplementary Figure 3.

**a.** Schematic diagram and target sequence of siRNAs specific for the back splicing of circRELL1. **b**. CircRELL1 expression and RELL1 RNA expression after treatment with two siRNAs via qRT-PCR. **c.** Results of circRELL1 levels in AGS and SGC-7901 cells transfected with circRELL1-overexpressing plasmid via qRT-PCR. **d.** CircRELL1 expression and RELL1 RNA expression after treatment with circRELL1 overexpression plasmid via qRT-PCR. Results are presented as mean ± SD of three independent experiments. *P < 0.05, **P < 0.01.

Supplementary Figure 4.

Alteration of circRELL1 affects the proliferation, migration and invasion. **a-d.** CCK-8 assay was utilized to measure proliferation of cells transfected with vector or circRELL1 plasmid, control or circRELL1 siRNAs. **e-j.** Edu assays were used to explore cell proliferation capability in GC cells (scale bar: 100 μm). **k-p.** Flow cytometry assays and TUNEL assays were conducted to access cell apoptosis rates in GC cells (scale bar: 100 μm). Results are presented as mean ± SD. *P < 0.05, **P < 0.01.

Supplementary Figure 5.

**a.** The assessment of the enrichment in Ago2 immunoprecipitates, as determined by WB. **b, c** qRT-PCR analysis of miR-637 expression after treatment with miR-637 controls, mimics and inhibitor. **d, e.** Kaplan–Meier analysis of the correlation between miR-637 expression and the overall survival (OS) and disease-free survival (DFS) of miR-637^hi^ and miR-637^lo^ patients. Log-rank tests were used to determine statistical significance. **f.** Transfection efficiency of miR-637-in and miR-637-mim. **P < 0.01, ***P < 0.001.

Supplementary Figure 6.

**a, b.** The miR-637 target genes levels in cells transfected with circRELL1 siRNA or miR-637 mimics, as qRT-PCR determined. **c.** Quantitative analysis of EPHB3 protein levels in GC samples. **d-f.** qRT-PCR of circRELL1, miR-637 and EPHB3 in tumors from the nude mice. **g.** Quantitative analysis of EPHB3 protein levels in AGS and SGC-7901 cells transfected with miR-637 inhibitor, miR-637 mimics and their control groups. **h, i.** IHC of EPHB3 in tumors of different treatments. **j.** Quantitative analysis of EPHB3 protein levels in AGS and SGC-7901 cells transfected with circRELL1 ov and their control groups. Results are presented as mean ± SD of three independent experiments. *P < 0.05, **P < 0.01, ***P < 0.001.

Supplementary Figure 7.

**a-b.** The levels of LC3-II/I in AGS and SGC-7901 cells co-cultured with exos-circRELL1 or treated with 3-MA (5 mM), rapamycin (100 nM), or control. **c-e.** Quantitative analysis of EPHB3, P62 and LC3II/I protein levels after co-transfection with circRELL1-ov, miR-637 mimics, or their controls. **f.** Kaplan–Meier analysis of the correlation between EPHB3 levels and survival probability based on the TCGA database. **g, h.** Kaplan–Meier analysis of the correlation between EPHB3 levels and progression-free survival (PFS) and OS based on the TCGA database. **i.** Transfection efficiency of EPHB3 ov and EPHB3 si. *p < 0.05, **p < 0.01.

Supplementary Figure 8.

The regulation of EPHB3 is mediated by miR-637. **a-h.** Results of CCK-8, colony formation, TUNEL assays and Edu assays were applied to measure the ability of proliferation after co-transfection with EPHB3 plasmid, miR-637 mimic, or controls (scale bar: 100 μm). **i-l.** The transwell assays were conducted to explore the cell migration and invasion capability under different treatments (scale bar: 100 μm). All data are presented as the mean ± SD. *P < 0.05, **P < 0.01.

Supplementary Figure 9.

Schematic diagram: circRELL1 promoted EPHB3 to repress GC proliferation, autophagy, migration, and invasion，thereby supressing the development of GC. Plasma exosomal circRELL1 may function as a potential diagnostic circulation biomarker in GC.
