# Supplementary figures and images for: Exosomal circRELL1 serves as a miR-637 sponge to modulate gastric cancer progression via regulating autophagy activation
Source: Cell Death Dis. 2022 Jan 13;13(1):56. doi: 10.1038/s41419-021-04364-6 (PMC8758736; doi:10.1038/s41419-021-04364-6)

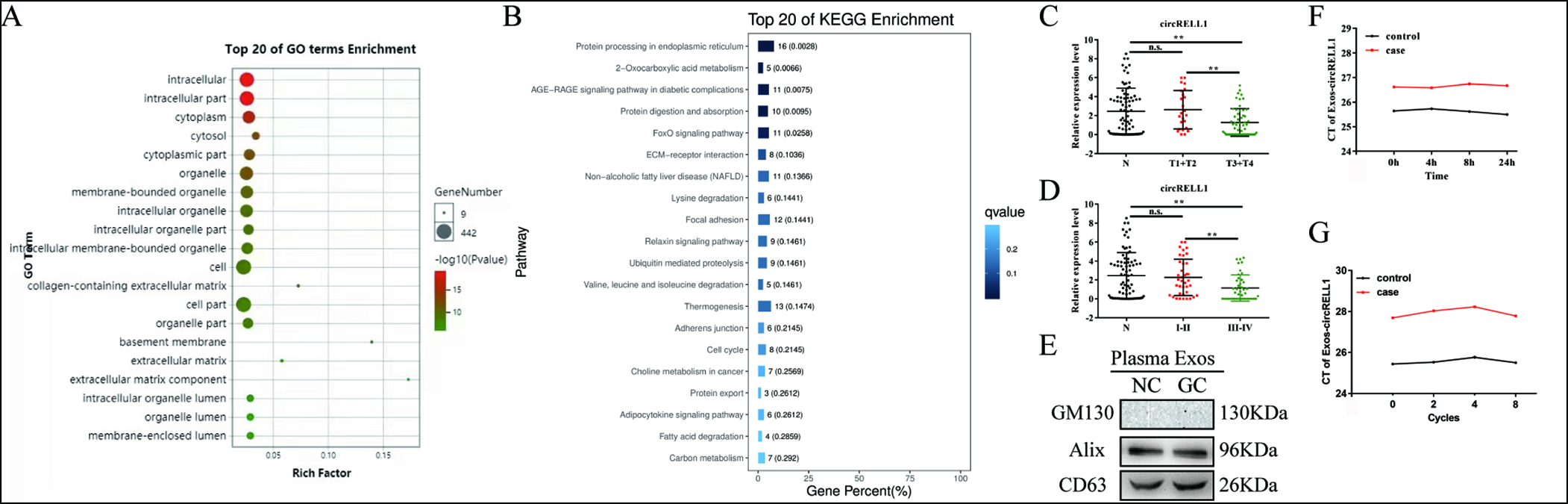

Supplement: Supplementary file 5 — Supplementary Figure 1 [file 41419_2021_4364_MOESM5_ESM.tif]

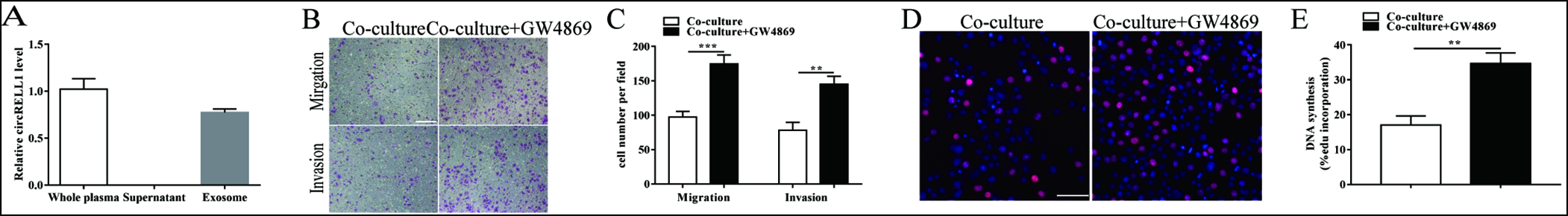

Supplement: Supplementary file 6 — Supplementary Figure 2 [file 41419_2021_4364_MOESM6_ESM.tif]

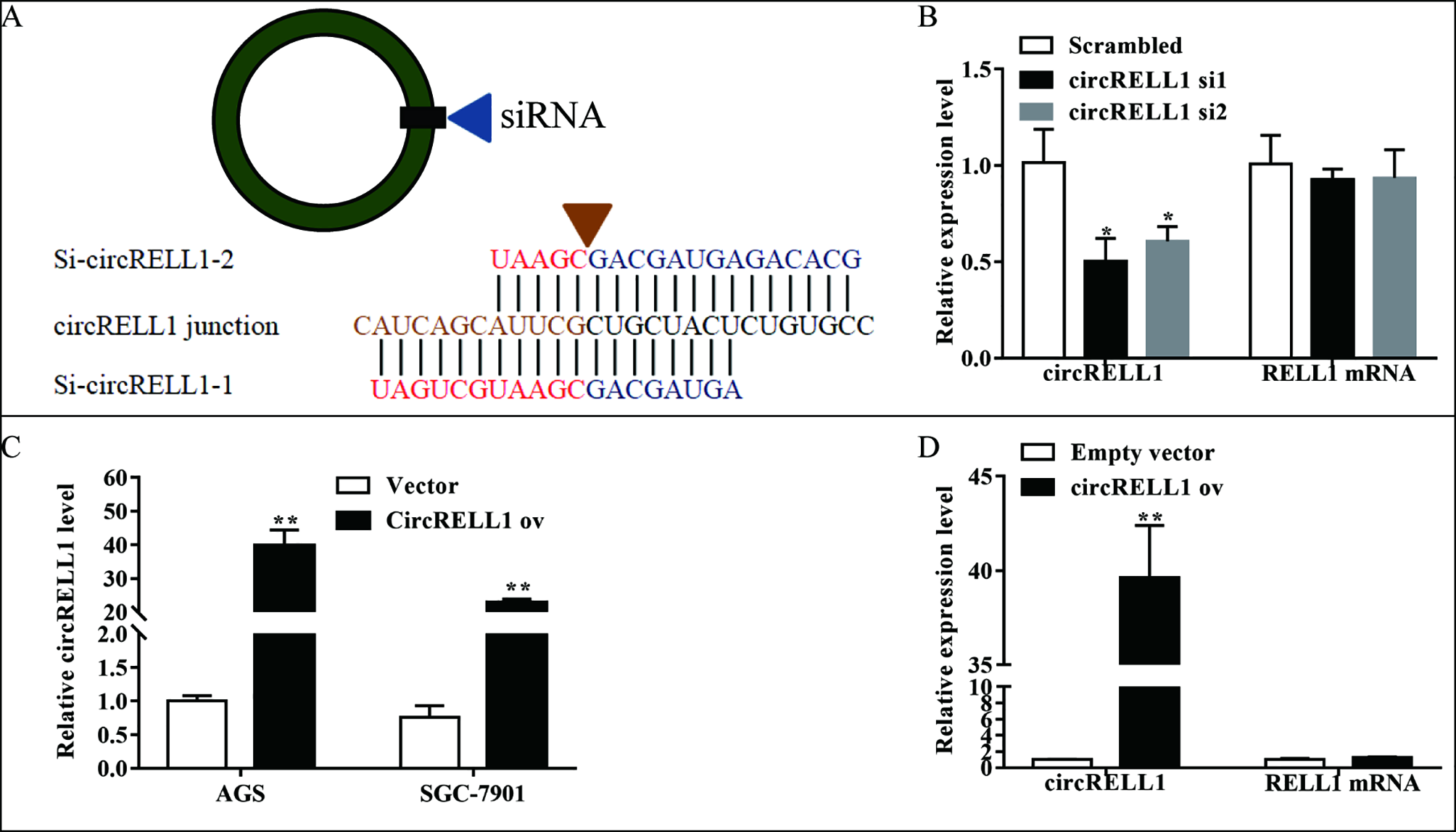

Supplement: Supplementary file 7 — Supplementary Figure 3 [file 41419_2021_4364_MOESM7_ESM.tif]

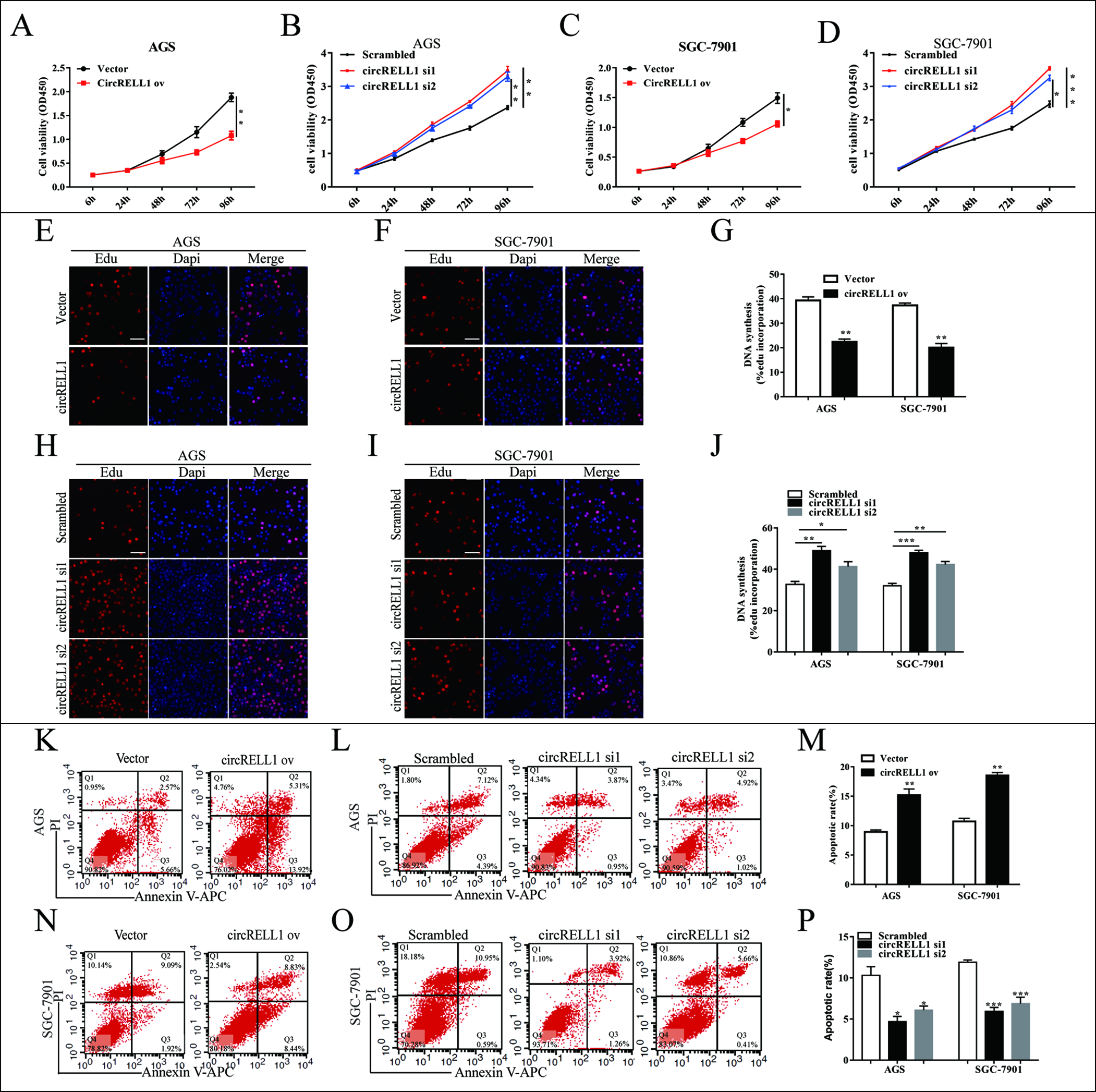

Supplement: Supplementary file 8 — Supplementary Figure 4 [file 41419_2021_4364_MOESM8_ESM.tif]

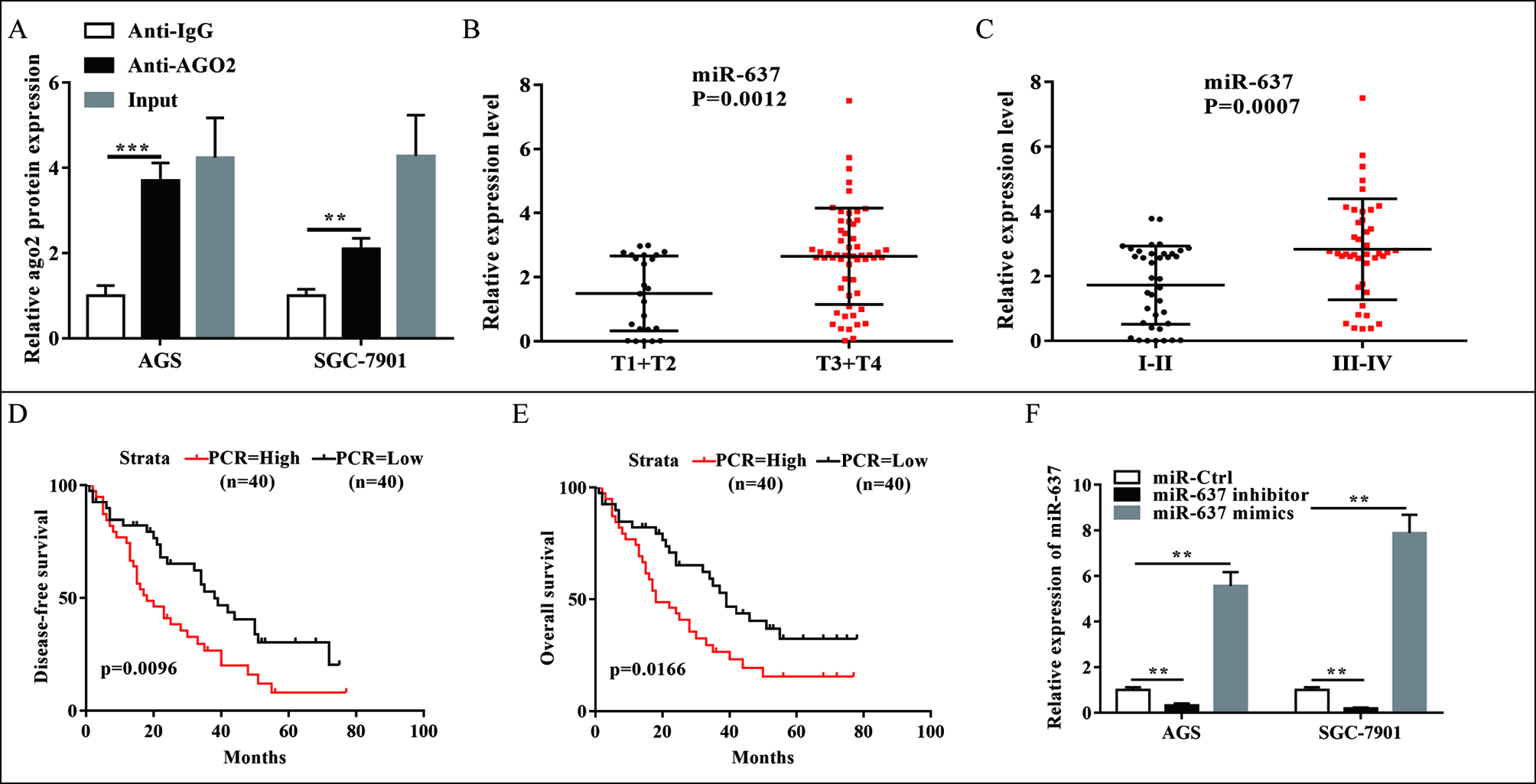

Supplement: Supplementary file 9 — Supplementary Figure 5 [file 41419_2021_4364_MOESM9_ESM.tif]

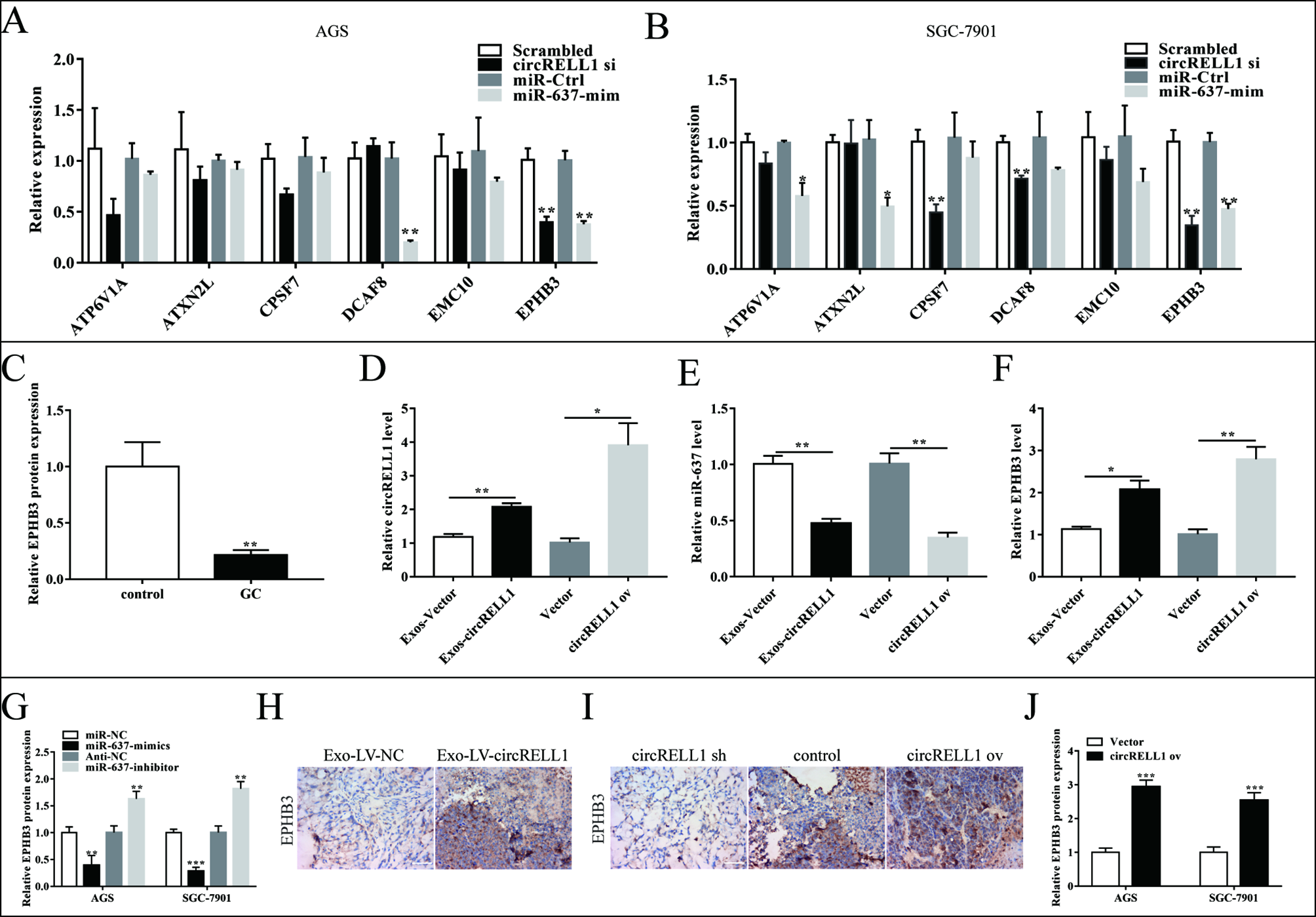

Supplement: Supplementary file 10 — Supplementary Figure 6 [file 41419_2021_4364_MOESM10_ESM.tif]

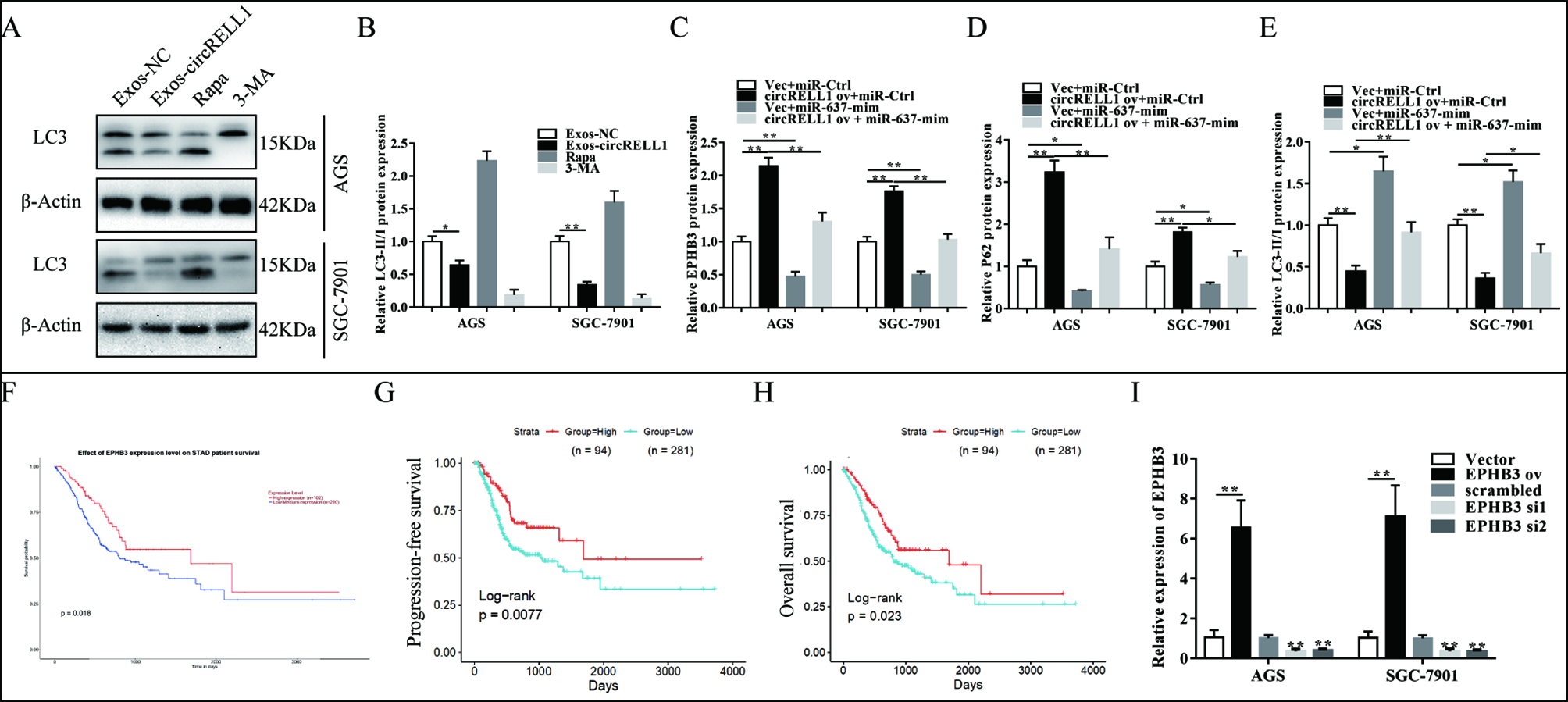

Supplement: Supplementary file 11 — Supplementary Figure 7 [file 41419_2021_4364_MOESM11_ESM.tif]

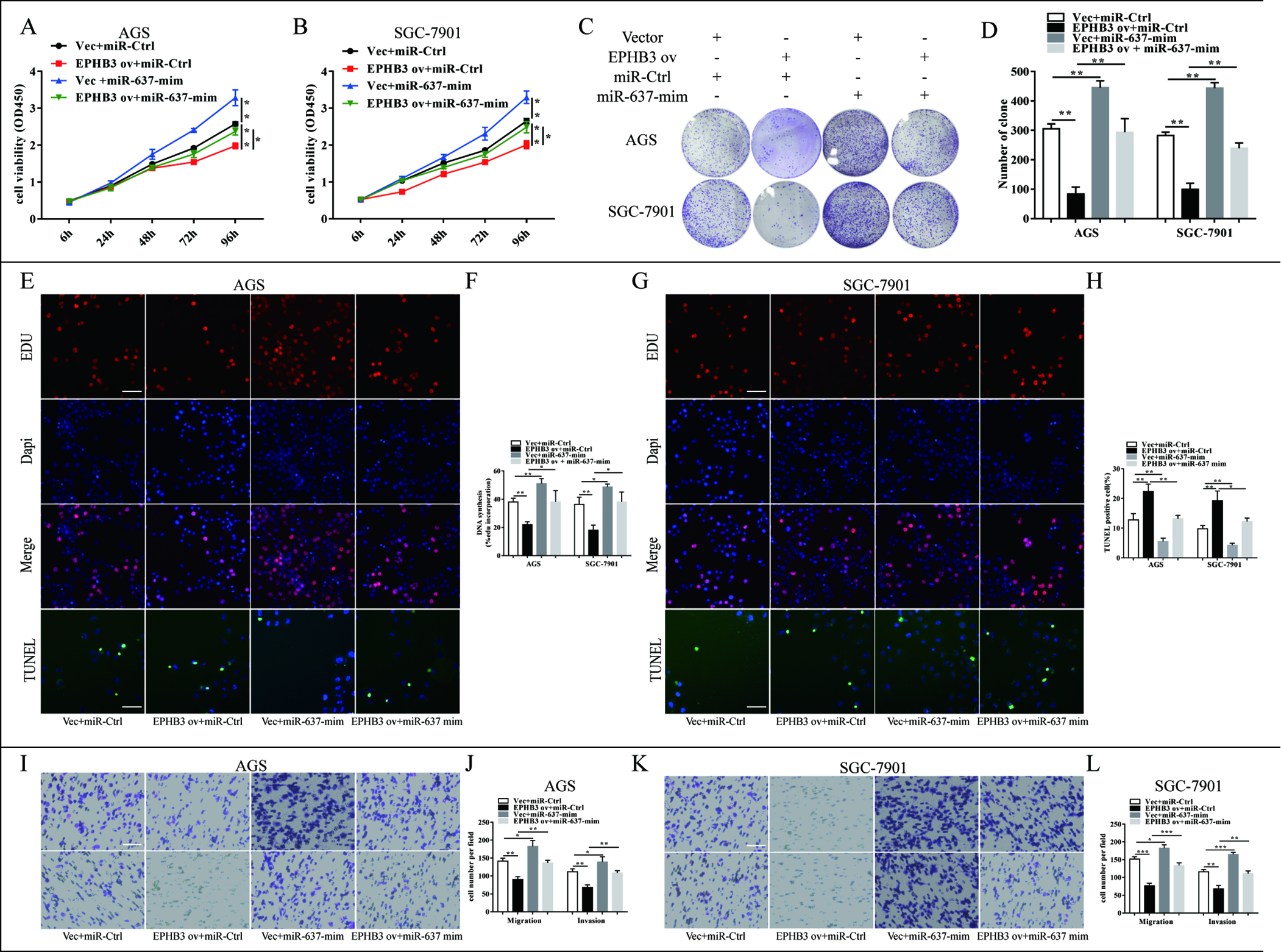

Supplement: Supplementary file 12 — Supplementary Figure 8 [file 41419_2021_4364_MOESM12_ESM.tif]

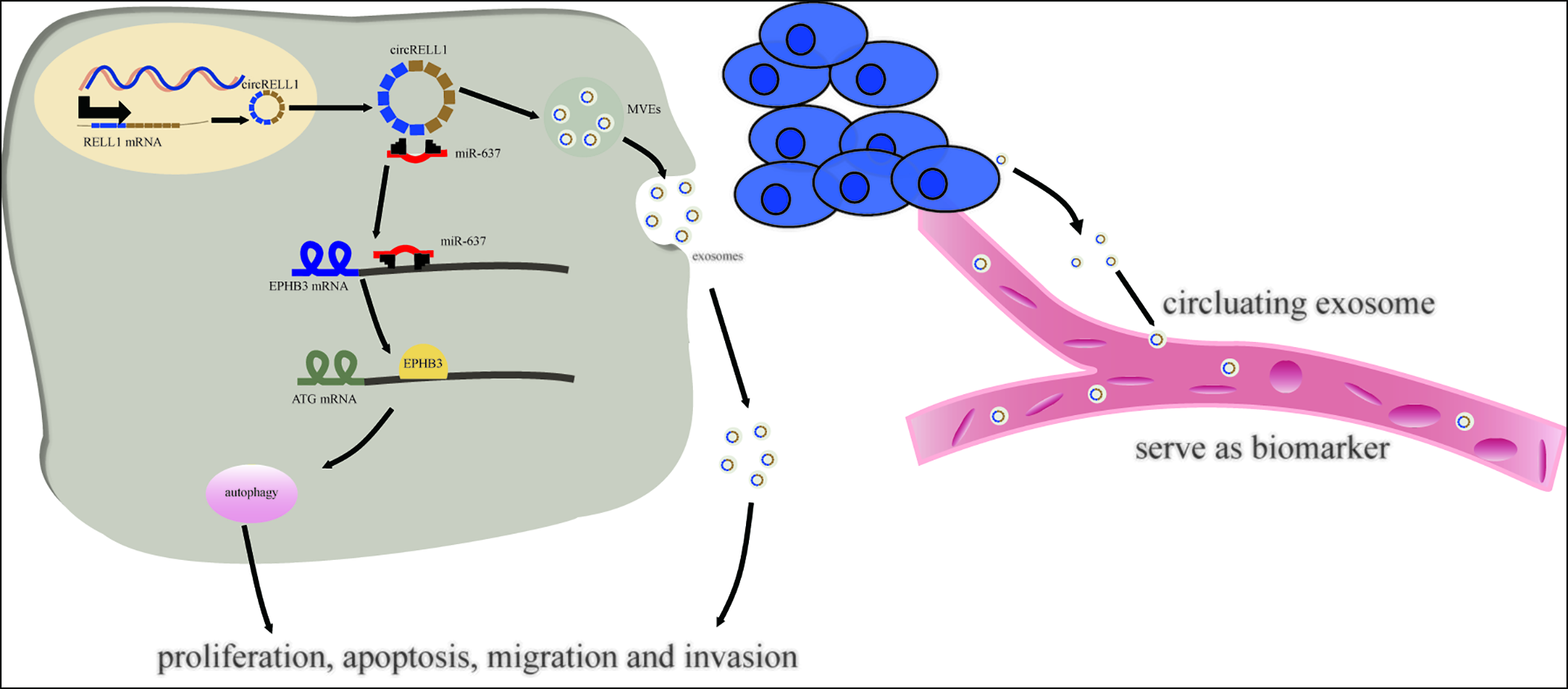

Supplement: Supplementary file 13 — Supplementary Figure 9 [file 41419_2021_4364_MOESM13_ESM.tif]
